# HR-Mamba: Building Footprint Segmentation with Geometry-Driven Boundary Regularization

**DOI:** 10.3390/s26020352

**Published:** 2026-01-06

**Authors:** Buyu Su, Defei Yin, Piyuan Yi, Wenhuan Wu, Junjian Liu, Fan Yang, Haowei Mu, Jingyi Xiong

**Affiliations:** 1 National Key Laboratory of Uranium Resource Exploration-Mining and Nuclear Remote Sensing, Beijing Research Institute of Uranium Geology, Beijing 100029, China; 2 Science and Technology on Near-Surface Detection Laboratory, The Fifth Research Institute of Wuxi, Wuxi 214035, China; 3 Institute of Desert Meteorology, China Meteorological Administration/National Observation and Research Station of Desert Meteorology, Taklimakan Desert of Xinjiang/Taklimakan Desert Meteorology Field Experiment Station of CMA, Urumqi 830002, China; 4 Xinjiang Key Laboratory of Desert Meteorology and Sandstorm, Urumqi 830002, China; 5 School of Geography and Ocean Science, Nanjing University, Nanjing 210023, China; 6 Urban Safety Engineering, Beijing Polytechnic College, Beijing 100042, China

**Keywords:** HR-Mamba, Mamba-SSM, geometry-driven post-processing, building semantic segmentation, high-resolution remote sensing

## Abstract

Building extraction underpins land-use assessment, urban planning, and disaster mitigation, yet dense urban scenes still cause missed small objects, target adhesion, and ragged contours. We present High-Resolution-Mamba (HR-Mamba), a high-resolution semantic segmentation network that augments a High-Resolution Network (HRNet) parallel backbone with edge-aware and sequence-state modeling. A Canny-enhanced, median-filtered stem stabilizes boundaries under noise; Involution-based residual blocks capture position-specific local geometry; and a Mamba-based State Space Models (Mamba-SSM) global branch captures cross-scale long-range dependencies with linear complexity. Training uses a composite loss of binary cross entropy (BCE), Dice loss, and Boundary loss, with weights selected by joint grid search. We further design a feature-driven adaptive post-processing pipeline that includes geometric feature analysis, multi-strategy simplification, multi-directional regularization, and topological consistency verification to produce regular, smooth, engineering-ready building outlines. On dense urban imagery, HR-Mamba improves F1-score from 80.95% to 83.93%, an absolute increase of 2.98% relative to HRNet. We conclude that HR-Mamba jointly enhances detail fidelity and global consistency and offers a generalizable route for high-resolution building extraction in remote sensing.

## 1. Introduction

With the rapid development of remote sensing technology, massive volumes of high-resolution imagery have demonstrated broad application value in resource surveys, environmental monitoring, military reconnaissance, and cartographic mapping. As key elements of urban space, buildings extracted from high-resolution imagery are crucial for land-use assessment, urban planning, and disaster prevention and mitigation. Nevertheless, in dense urban scenes with complex backgrounds, achieving accurate region-level segmentation and fine-grained contour delineation while preserving details remains a central challenge in remote sensing image understanding [1,2].

Traditional building extraction methods primarily rely on low-level cues such as texture, shape, and color. Representative techniques include object-based approaches, thresholding, auxiliary-information fusion, and machine-learning-based classification [3]. In object-based image analysis, the image is segmented into objects and then classifies them using spectral and shape features [4,5]; thresholding separates foreground and background on statistical criteria [6]; and auxiliary-information methods exploit multi-source data (e.g., LiDAR point clouds and shadow priors) to enhance robustness [7,8,9]. Under the machine learning paradigm, hand-crafted spectral and texture descriptors are commonly fed into classifiers such as support vector machines (SVMs) [10,11], random forests [12], and boosting algorithms [13] for building or non-building discrimination. Although these pipelines offer a degree of interpretability, they are sensitive to hyperparameters and scene conditions and often degrade in dense urban scenes, particularly for small or closely spaced buildings and precise footprint geometry.

In recent years, driven by advances in computing hardware and the rise of deep learning [14,15], end-to-end, data-driven learning has achieved significant progress in remote sensing image interpretation. Convolutional neural networks (CNNs) [16,17,18] learn high-level semantic representations from large annotated datasets and have become the mainstream paradigm for high-resolution imagery [19,20]. For building extraction, early studies employed CNN, Faster R-CNN-style detectors for localization and recognition, verifying the feasibility of deep models for this task [21,22,23,24]. Subsequently, semantic segmentation networks such as FCN, SegNet, and DeepLabV3+ have substantially improved pixel-level accuracy through encoder–decoder designs and multi-scale feature fusion [25,26,27]. By maintaining high-resolution representations with parallel multi-resolution branches, High-Resolution Network (HRNet) has become a widely used backbone for building semantic and instance segmentation [28]. More recently, vision Transformers (e.g., Swin Transformer, SegFormer) leverage hierarchical self-attention and lightweight decoders to strengthen global context and cross-scale dependencies, achieving strong performance on remote sensing segmentation [29,30]; however, boundary refinement and small-object preservation still rely on sufficient spatial resolution and fine-grained features. By contrast, selective state space models (SSMs) represented by Mamba establish long-range dependencies with Selective Scan at linear complexity *O*(*L*), offering superior memory efficiency and throughput compared to self-attention’s *O*(*L*^2^). Recently, Vision-Mamba-based and Mamba-based networks such as CVMH-UNet and MFMamba have already demonstrated strong performance on remote sensing semantic segmentation, including high-resolution building extraction scenarios [31,32], further highlighting the potential of Mamba-style SSMs for large-scale, detail-sensitive mapping tasks [33].

On the other hand, deep segmentation networks usually output raster masks; naive vectorization tends to introduce aliasing, spurs, and topological errors, which conflict with the geometric constraints required in geographic information system (GIS) cartography and mapping [34]. Geometry-driven contour regularization therefore becomes a critical step. Typical strategies include Douglas–Peucker (DP) [35] based polygon simplification and fine polygon regularization (FPR) [36], which enforce linear and orthogonal direction constraints and edge alignment to achieve a balance between morphological fidelity and geometric regularity.

Despite the overall accuracy gains of deep learning, limitations persist in capturing subtle structures: small buildings are prone to false negatives or false positives, and boundaries between adjacent buildings often adhere (“stick”), degrading segmentation accuracy and shape consistency. Meanwhile, naive vectorization frequently yields irregular building outlines, hindering direct use in geospatial analysis [37]. Accordingly, simultaneously enhancing boundary-detail representation and the geometric usability of vector contours remains a core problem.

To address these issues, we propose High-Resolution-Mamba (HR-Mamba), an independently developed framework for building footprint segmentation (distinct from the HRMamba implementation on GitHub, https://github.com/kevinaza111/HRMamba, accessed on 1 November 2025). HR-Mamba integrates an edge-aware stems, Involution-based residual blocks, Mamba-based state space modeling, and geometry-driven regularization into a high-resolution design centered on detail preservation and efficient long-range dependency modeling. The network is trained with a composite objective that combines binary cross entropy (BCE), Dice, and Boundary losses, whose weights are selected by joint grid search, and is coupled with a geometry-driven vector pipeline that includes DP simplification, fine polygon regularization, directional clustering, and topological validation. Experiments demonstrate that, in complex urban scenes, the proposed method markedly reduces missed small targets and adhesion between neighboring buildings and produces clearer boundaries with more regular polygons.

## 2. Methods

**Figure 1:**
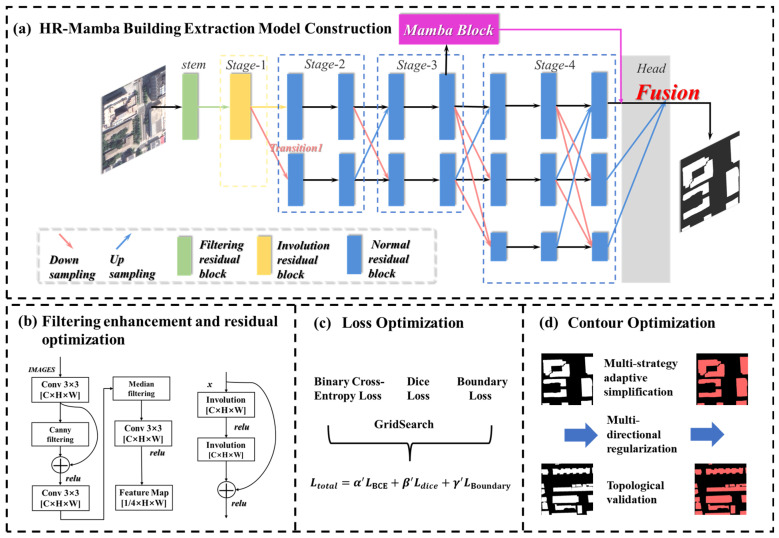
Research framework for building extraction based on the HR-Mamba model.

Figure 1 presents the overall workflow of the proposed HR-Mamba framework. Based on this workflow, the subsequent subsections describe each component in detail. Section 2.1 introduces the HR-Mamba network architecture; Section 2.2 explains the filtering-enhanced, edge-aware stem; Section 2.3 presents the Involution-based residual block for local geometric modeling; Section 2.4 describes the Mamba-SSM global branch for modeling long-range dependencies; Section 2.5 formulates the composite loss function; and Section 2.6 develops the geometry-driven post-processing strategy for generating regular building polygons.

As shown in Figure 1, the building extraction framework based on HR-Mamba follows a sequence of low-level boundary stabilization, shallow-level geometry, high-level global modeling, loss synergy, and geometric regularization. As illustrated in Figure 1a,b, we incorporate Canny [38] edge enhancement and median filtering [39] in the stem to improve boundary accuracy and noise robustness. In Stage 1, an improved Involution [40] residual block models local geometric features. In Stage 3, a Mamba-SSM global branch captures cross-scale long-range dependencies, enabling synergy between local details and global semantics. As shown in Figure 1c, a composite loss comprising BCE loss [41], Dice loss [25], and Boundary loss [42] is used to mitigate foreground and background imbalance and to strengthen boundary learning. Finally, as illustrated in Figure 1d, a geometry-driven adaptive post-processing strategy is applied to the network outputs for contour simplification, regularization, and topological verification, yielding fine-grained, regular, and robust building segmentation results.

### 2.1. HR-Mamba Network Architecture

**Figure 2:**
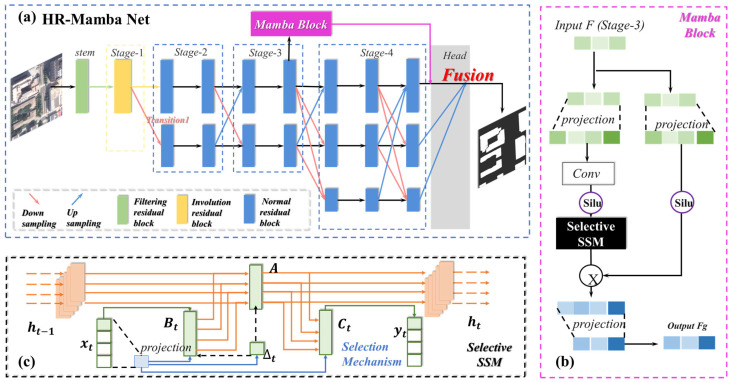
HR-Mamba Network Architecture. (**a**) Overall architecture of the HR-Mamba network. (**b**) Structure of the Mamba block inserted at Stage 3. (**c**) Selective state space model (Selective SSM) module within the Mamba block.

HR-Mamba is a high-precision semantic segmentation model for remote sensing imagery, and its architecture is illustrated in Figure 2a. For clarity, we summarize its main components below. Building on the classic HRNet multi-resolution parallel design, HR-Mamba introduces targeted enhancements at two levels: low-level feature extraction and high-level global modeling.

(1) Local edge enhancement. In the stem, we embed a lightweight filtering module that couples convolutional image features with filtering to improve the robustness and noise resistance of the initial representation, laying a stable foundation for subsequent multi-resolution feature extraction. Specifically, Canny operators are used to extract edges and are added as a residual enhancement, and a median filter is then applied to smooth spurious noise, which highlights building boundaries while suppressing background interference. Unlike stochastic data augmentation, which perturbs raw images only during training, this stem-level filtering is applied deterministically to early feature maps at both training and inference time, so that the Canny and median operations provide a stable, edge-aware, and denoised representation that later HRNet stages can consistently build upon.

(2) Shallow local modeling. In Stage 1, Involution-based residual blocks replace the standard residual units in HRNet. Involution is a position-specific variant of convolution that generates adaptive kernels for each spatial location, thereby strengthening the modeling of spatial details. Introducing Involution residual blocks enables effective extraction of fine-grained geometric cues, for example, corners, narrow gaps, and intricate edges, improving the fidelity of local feature representations. The intermediate Stage 2 retains the standard HRNet configuration without modification and is therefore detailed here for brevity.

(3) High-level global representation. In Stage 3, a Mamba-SSM-based global selective state space branch is incorporated to enhance cross-scale long-range dependency modeling. Through a selective gating mechanism, this branch establishes global feature dependencies while retaining only the contextual information that is informative for segmentation. Combined with the parallel multi-scale fusion of HRNet, the global features introduced by Mamba-SSM complement high-resolution local features, allowing the network to preserve local details and maintain global consistency simultaneously.

In summary, HR-Mamba preserves the strengths of HRNet in multi-resolution parallel representation and augments them with local filtering enhancement and global sequence modeling. This combination achieves a balanced treatment of boundary-detail preservation and global semantic consistency for building segmentation.

### 2.2. Filtering-Enhanced Module (Stem Stage)

At the input pre-processing stage we embed a cascaded filtering-enhanced structure into the HRNet stem to highlight building boundary details and suppress noise. We denote this design as the Canny-with-median stem (CM_stem).

Building on our earlier study of stem-level edge and denoising strategies on an HRNet backbone, we introduce a dedicated stem variant, CM_stem, which embeds Canny edge augmentation and median filtering into the HRNet stem and achieves the best balance of accuracy and robustness. In CM_stem, the stem first extracts an initial feature map $F_{0}$ with a 3 × 3 convolution and then injects Canny responses in a residual manner to accentuate salient contours [43]. A channel-reducing projection is subsequently applied, followed by median filtering to attenuate stray noise and pseudo-edge responses. The enhanced features are then passed to strided convolutions as the initial representation for multi-resolution feature extraction. This design preserves boundary integrity while improving feature robustness and separability, providing stable support for cross-scale representation. Below we briefly summarize the two main steps.

(1) Initial feature extraction and edge detection.

Given an input image $I\left(x,y\right)$, an initial feature map $F_{0}$ is produced by convolution:

(1) $$F_{0}=Conv_{3\times 3}\left(I\right),\;\;\;F_{0}\in R^{C\times H\times W}$$

A standard Canny edge detector is then applied to $F_{0}$ to obtain a binary edge map $E\left(x,y\right)$, including Gaussian smoothing, gradient computation, non-maximum suppression, and double-threshold hysteresis. Since these steps follow the classical Canny formulation, we do not repeat their equations here and instead refer the reader to [38] for details. In all experiments, the Gaussian smoothing parameter σ as well as the high and low hysteresis thresholds of the Canny operator are selected once on the validation set and then kept fixed for all images, rather than being re-tuned for each image.

The edge map is residually fused with the initial features and activated:

(2) $$F_{1}=ReLU\left(F_{0}+E\right)$$

followed by an additional convolution to obtain enhanced features:

(3) $$F_{2}=Conv_{3\times 3}\left(F_{1}\right)$$

(2) Median filtering and feature mapping.

On top of the edge-enhanced features $F_{2}$, a median filter further suppresses isolated noise while preserving edges:

(4) $$F_{3}=Median\{F_{2}\left(i,j\right)|\left(i,j\right)\in \Omega \left(x,y\right)\}$$

where $\Omega \left(x,y\right)$ denotes a local neighborhood (e.g., 3 × 3 or 5 × 5). Concretely, a sliding window is centered at each pixel; all intensities within the window are sorted, the middle value is selected, and the center pixel is replaced by this median, thereby reducing impulse noise without eroding edge structures.

Finally, two strided (stride = 2) convolutional layers are applied to progressively downsample the features, yielding the stem output:

(5) $$F_{stem}=ReLU\left(Conv_{3\times 3,s=2}\left(Conv_{3\times 3,s=2}\left(F_{3}\right)\right)\right),\;\;F_{stem}\in R^{\frac{C}{8}\times \frac{H\times W}{4}}$$

### 2.3. Involution Residual Block

**Figure 3:**
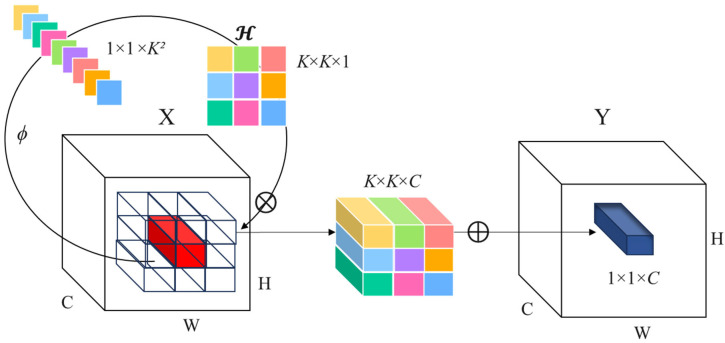
Computational structure of Involution.

Involution is a novel variant of convolution proposed by Li et al [40]. Whose design philosophy differs from standard convolution and depthwise convolution. Traditional convolution shares kernels across spatial positions and is position-agnostic along the channel dimension; Involution inverts this: it is position-specific in the spatial domain while sharing kernels across channels. This design allows the operator to flexibly model the spatial distribution of input features. Its structure is illustrated in Figure 3. Intuitively, this makes Involution particularly suitable for capturing spatially varying building shapes and fine details.

Concretely, for each spatial position $\left(i,j\right)$ of the input feature map, Involution generates a dedicated kernel that is shared among channels within a group, and the output is computed via multiply–accumulate operations:

(6) $$Y_{i,j,k}=\sum_{\left(u,v\right)\in \Delta K}H_{i,j,u+\lfloor K/2\rfloor ,v+\lfloor K/2\rfloor ,\lfloor kG/C\rfloor }X_{i+u,j+v,k}$$

where $\left(i,j\right)$ indexes spatial positions, *k* indexes channels, ΔK denotes the spatial support of the kernel, *C* is the number of channels, and *G* is the number of channel groups.

Unlike standard convolution, the kernel shape of Involution depends on the input and is adaptively generated from the input features:

(7) $$H_{i,j}=\Phi \left(X_{\psi_{i,j}}\right)=W_{1}\sigma \left(W_{0}X_{i,j}\right)$$

where $\Phi \left(\cdot \right)$ is a lightweight kernel generator typically composed of an MLP, normalization, and an activation function; $W_{0}$ and $W_{1}$ are linear transforms forming a bottleneck to reduce intermediate dimensionality; and $\sigma \left(\cdot \right)$ denotes the non-linear activation (applied after batch normalization) for the two linear transforms. With this design, each spatial location obtains an input-adaptive kernel, which retains the efficiency of channel sharing while enhancing spatial detail modeling and the flexibility of feature expression. As a result, Involution adaptively emphasizes distinct local building patterns, such as corners, gaps between structures, or elongated edges of building roofs, according to the surrounding contextual information.

Therefore, Involution can be viewed as a lightweight, adaptive convolutional variant that more effectively captures building boundaries and geometric details in complex scenes, making it well-suited for the proposed residual block replacement in Stage 1.

**Figure 4:**
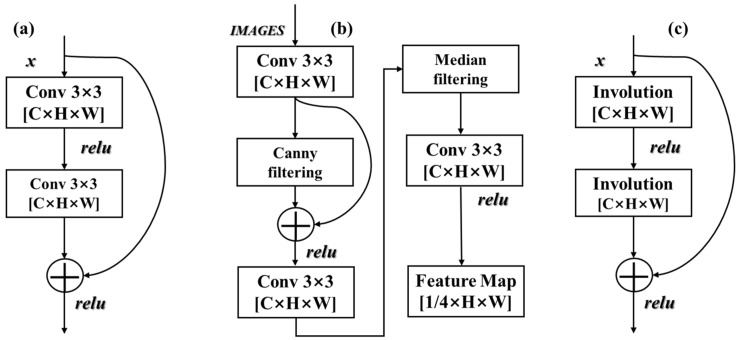
Residual block variants: (**a**) standard convolutional residual block; (**b**) depthwise-separable convolution residual block; (**c**) Involution residual block.

In this study, we embed the Involution convolutional residual block, as illustrated in Figure 4c, into the network to replace the standard residual unit in Stage 1 of HRNet, as shown in Figure 4a. This modification preserves the model’s high-resolution representational capacity and enables more effective capture of fine-grained geometric features, including building corners, narrow gaps, and elongated boundaries, thereby markedly improving structural refinement in the building extraction process.

### 2.4. Mamba-SSM Global Branch

Although the filtering-enhanced stem and the Involution blocks strengthen the ability of HR-Mamba to capture local edges and geometric details, purely local convolutional operators struggle to model globally consistent semantics when wide-range context is required, for example, shadow cast, adhesion among multiple adjacent buildings, and elongated buildings that span image boundaries. Following the standard Mamba-SSM formulation [33], we adopt an off-the-shelf Mamba-SSM module and focus on integrating it as a global branch into a high-resolution HRNet backbone for building footprint segmentation. In this subsection, we briefly describe how this branch is connected to Stage 3 features and how it contributes to global context modeling.

To address this limitation, on top of the Stage 3 feature representation of HRNet, an additional Mamba-SSM global selective state space branch is introduced, as illustrated in Figure 2a, to enhance cross-scale long-range dependency modeling and improve global contextual consistency. The internal structure of the Mamba-SSM module is depicted in Figure 2b.

As illustrated in Figure 2b, the Mamba block takes the Stage 3 features $F_{S3}$ as input. Two projection paths are used to perform channel alignment and feature compression. We first flatten $F_{S3}$ into a 1D sequence of length L=H×W using a row-major (raster-scan, top-to-bottom and left-to-right) ordering, and the Mamba kernel operates along this sequence. Two 1 × 1 convolutional projection paths are then used for channel alignment and feature compression: the main branch reduces the channel dimension from C to $C_{m}=C/2$, applies a SiLU activation, feeds the result into the selective SSM kernel, and finally projects it back to C channels, whereas the bypass branch keeps C channels, applies SiLU, and is added residually. Within the selective SSM kernel, we follow the standard Mamba configuration and use depthwise 1D convolutions with a small kernel size (k=3) along the flattened spatial sequence to parameterize the state transitions. For input images of size 500 × 500, the Stage 3 feature map in our configuration has a spatial resolution of 125 × 125 with 256 channels.

Within the selective SSM kernel, as shown in Figure 2c, the hidden state is updated in an input-dependent manner. Its discrete form is:

(8) $$x_{t+1}=A\left(\Delta_{t}\right)\cdot x_{t}+B\left(\Delta_{t}\right)\cdot u_{t},y_{t}=C\left(\Delta_{t}\right)\cdot x_{t}+D\left(\Delta_{t}\right)\cdot u_{t}$$

where $x_{t}$ denotes the hidden state at step *t*, $u_{t}$ denotes the input sequence features, and $y_{t}$ the output features; the matrices *A*, *B*, *C*, *D* adapt with the input step $\Delta_{t}$. This design maintains stability while adapting to variations in spatial resolution and semantic complexity within the feature representations.

Through the Selective Scan mechanism, the SSM establishes long-range dependencies over the entire spatial extent while a selective gating suppresses propagation from redundant regions, retaining only context informative for segmentation. This is particularly effective under shadow interference or adhesion of neighboring buildings, ensuring boundary consistency and continuity. Compared with the *O*(*L^2^*) complexity of Transformer-style global self-attention, Mamba’s Selective Scan operates in linear time *O*(*L*), offering a marked efficiency advantage for large-scale remote sensing imagery.

The SSM kernel output is then added pointwise to the bypass projection (residual), forming a residual-enhanced structure; normalization is applied to maintain numerical stability. A final projection maps the result to the globally enhanced features $F_{g}$, which constitute the output of the Mamba block. This output preserves sensitivity to local geometry while imposing global consistency constraints, thus providing a higher-level representation for the subsequent head fusion.

In the head fusion layer, the global features $F_{g}$ are fused with the multi-scale features $F_{hr}$ from the HRNet backbone using a weighted scheme:

(9) $$F_{fusion}=\lambda F_{hr}+\left(1-\lambda \right)F_{g},\;\lambda \in \left[0,1\right]$$

where the fusion coefficient λ is automatically selected on the validation set during training rather than set manually. This mechanism adaptively balances local geometric detail and global contextual consistency across scenes, thereby markedly improving robustness and fine-grained quality of building segmentation in complex urban environments. Moreover, compared with the HRNet baseline under the given input configuration, incorporating the Mamba-SSM branch adds only 156,912 parameters and approximately 0.26 GFLOPs, corresponding to merely about 0.24% more parameters and 0.28% more computation, indicating minimal overhead.

### 2.5. GridSearchCombinedLoss: Construction and Optimization

In building semantic segmentation, a single loss rarely balances global pixel accuracy, class imbalance, and boundary detail simultaneously. We therefore adopt a composite objective, denoted GridSearchCombinedLoss, which linearly fuses BCE loss, Dice loss, and Boundary loss with learnable weights. By loss-scale normalization and a grid-search strategy over the weight space, the optimal combination is automatically selected, achieving a dynamic equilibrium among multiple objectives.

(1) BCE loss.

BCE loss provides pixelwise supervision by measuring the discrepancy between predicted probabilities and ground-truth labels:

(10) $$L_{BCE}=-\frac{1}{N}\sum_{i=1}^{N}\left(y_{i}\log p_{i}+\left(1-y_{i}\right)\log\left(1-p_{i}\right)\right)$$

where $p_{i}\in \left[0,1\right]$ is the predicted probability for pixel i and $y_{i}\in \left[0,1\right]$ denotes the true label of pixel i. Its gradient is:

(11) $$\frac{\partial L_{BCE}}{\partial p_{i}}=\{\begin{array}{c} -\frac{1}{p_{i}},y_{i}=1\\ \frac{1}{1-p_{i}},y_{i}=0 \end{array}$$

When $y_{i}=1$, the negative gradient drives $p_{i}$ toward 1, emphasizing correct classification of foreground pixels; when $y_{i}=0$, the positive gradient drives $p_{i}$ toward 0, strengthening background suppression. Accordingly, BCE provides stable global pixel-level supervision for the segmentation.

(2) Dice loss.

Dice loss directly optimizes the overlap between prediction and ground truth and is more robust under foreground sparsity or class imbalance:

(12) $$L_{dice}=1-\frac{2\sum_{i=1}^{N}y_{i}\cdot p_{i}}{\sum_{i=1}^{N}y_{i}+\sum_{i=1}^{N}p_{i}}$$

Here, $\overset{N}{\underset{i=1}{\sum }}y_{i}\cdot p_{i}$ denotes the intersection, while $y_{i}$ and $p_{i}$ denote the sizes of the ground-truth and predicted regions, respectively, and *N* is the number of pixels. For *i* = 1, the gradient $p_{1}$ is:

(13) $$\frac{\partial L_{dice}}{\partial p_{1}}=\{\begin{array}{c} \frac{2\sum_{i=1}^{N}y_{i}p_{i}}{{\left(\sum_{i=1}^{N}y_{i}+\sum_{i=1}^{N}p_{i}\right)}^{2}},y_{1}=0\\ -\frac{2\left(\sum_{i=1}^{N}y_{i}+\sum_{i=1}^{N}p_{i}\right)-2\sum_{i=1}^{N}y_{i}\cdot p_{i}}{{\left(\sum_{i=1}^{N}y_{i}+\sum_{i=1}^{N}p_{i}\right)}^{2}},y_{1}=1 \end{array}$$

From the above, when $y_{1}=0$, the gradient is positive, guiding the network toward ${\hat{y}}_{i}=0$ and thus focusing more on background pixels; when $y_{1}=1$, the gradient is negative, guiding ${\hat{y}}_{i}=1$ and thereby improving attention to foreground pixels.

(3) Boundary loss.

Boundary loss introduces a distance-transform weighting to strengthen learning near object boundaries:

(14) $$L_{Boundary}=-\frac{1}{N}\sum_{i=1}^{N}d\left(y_{i}\right)\cdot \left|p_{i}-y_{i}\right|$$

where $d\left(y_{i}\right)\geq 0$ denotes the unsigned Euclidean distance from pixel to the ground-truth boundary. Its gradient is:

(15) $$\frac{\partial L_{Boundary}}{\partial p_{i}}=\{\begin{array}{c} d\left(y_{i}\right),p_{i}>y_{i}\\ -d\left(y_{i}\right),p_{i}<y_{i} \end{array}$$

Hence, pixels near the boundary (for which $d\left(y_{i}\right)$ is smaller) have relatively amplified gradients, driving the predicted contour to approach the true boundary. Boundary loss improves boundary consistency and geometric accuracy.

(4) GridSearchCombinedLoss.

For building segmentation, the proposed GridSearchCombinedLoss exploits the complementary strengths of BCE, Dice, and Boundary losses to jointly optimize pixel accuracy, class balance, and boundary continuity. The intuition is: BCE supplies stable pixelwise supervision for overall reliability; Dice boosts recall in sparse foreground regions and mitigates class imbalance; Boundary enforces geometric consistency so that contours are more continuous and regular. Their spatial roles are complementary: background regions rely mainly on BCE, foreground interiors on Dice, and boundary details on Boundary.

Combining the three terms, the total loss is:

(16) $$L_{total}=\alpha L_{BCE}+\beta L_{dice}+\gamma L_{Boundary}$$

where α, β, and γ are weights that balance the contributions of the three components. In practice, we first normalize the scales of the three losses so that their magnitudes are comparable and then restrict the search to α ∈ (0.4, 0.5, 0.6), β ∈ (0.2, 0.3, 0.4), γ ∈ (0.0, 0.1, 0.2) with α+β+γ=1. This setting is motivated by the observation that the normalized Boundary loss is usually smaller than BCE and Dice, so keeping γ in a moderate range allows the boundary term to act as a regularizer without dominating the optimization. A grid search over this space is then used to identify the triplet (α, β, γ) that minimizes the validation loss.

With this multi-objective weighting scheme, the BCE term provides stable pixel-level supervision, the Dice term improves recall for building regions under class imbalance, and the Boundary term enhances contour precision. These terms are complementary, yielding a balanced trade off among overall accuracy, class balance, and boundary detail. Experiments on our dataset confirm the effectiveness of the composite objective: relative to a single loss baseline, the proposed combined loss substantially increases the detection rate of small buildings and improves boundary localization accuracy. Detailed comparisons are provided in subsequent sections.

Overall, GridSearchCombinedLoss allows HR-Mamba to simultaneously improve global pixel accuracy, foreground recall, and boundary precision without changing the network architecture.

### 2.6. Adopt Post-Processing Strategy for Buildings

**Figure 5:**
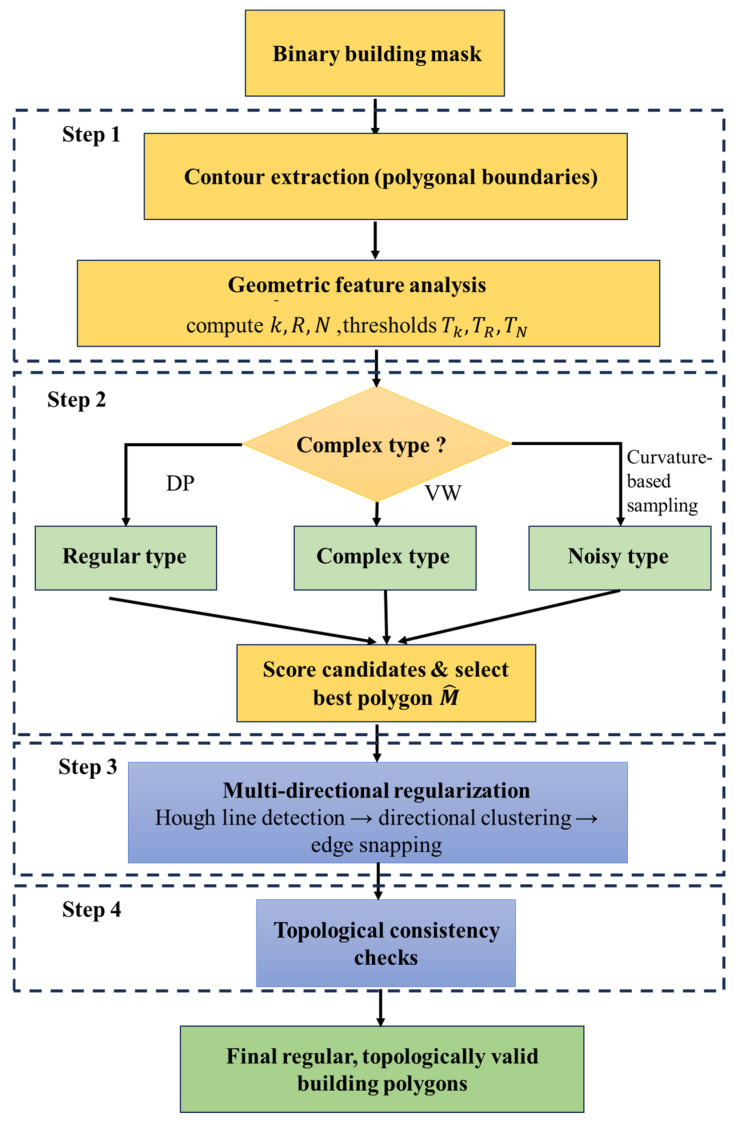
Geometry-driven adaptive post-processing pipeline for building polygons.

Deep-learning-based segmentation networks typically output building masks whose contours are influenced by model resolution, convolutional sampling, and noise, often resulting in aliasing, spurs, and irregular boundaries. To convert these masks into geometrically regular and topologically consistent building polygons, we design a geometry-driven adaptive post-processing framework, summarized in Figure 5. Concretely, the pipeline comprises four steps: geometric feature analysis, multi-strategy adaptive simplification, multi-directional regularization, and topological consistency checks. These steps preserve morphological fidelity while ensuring engineering usability.

In the first step (geometric feature analysis), given an extracted building polygonal mask contour $M={\left\{\left(x_{i},y_{i}\right)\right\}}_{i=1}^{n}$ denoting the *i* vertex, we compute three classes of geometric characteristics to measure contour complexity and noise level.

Local curvature:

(17) $$k_{i}=\frac{\left|{\vec{v}}_{i-1}\times {\vec{v}}_{i}\right|}{\left|{\vec{v}}_{i-1}\right|\cdot \left|{\vec{v}}_{i}\right|},{\vec{v}}_{i-1}=M_{i}-M_{i-1},{\vec{v}}_{i}=M_{i+1}-M_{i}$$

Here, $k_{i}$ is the discrete curvature at vertex iii, reflecting the local turning of the boundary; $\bar{k}$ (the mean curvature over all vertices) describes overall smoothness.

Perimeter–area ratio:

(18) $$R=\frac{P}{A}=\frac{\sum_{i}\sqrt{{\left(x_{i+1}-x_{i}\right)}^{2}+{\left(y_{i+1}-y_{i}\right)}^{2}}}{Area\left(M\right)}$$

A larger R indicates a more corrugated, complex boundary.

Smoothed points and noise level. For each vertex $m_{i}$, a locally weighted smoothing is applied:

(19) $$\tilde{m_{i}}=\frac{\sum_{j}w_{j}m_{i+j}}{\sum_{j}w_{j}},w_{j}=\frac{1}{\left|j\right|+1}$$

Here, $\tilde{m_{i}}$ is the smoothed point; $m_{i+j}$ are neighbors indexed by offset *j*; $w_{j}$ are the corresponding weights. The global noise level is then defined as:

(20) $$N=\frac{1}{n}\sum_{i=1}^{n}\frac{d_{i}}{P},d_{i}=\|m_{i}-\tilde{m_{i}}\|$$

where *N* quantifies global noise intensity; *n* is the number of vertices; $d_{i}$ is the Euclidean deviation between original and smoothed points; *P* is the contour perimeter.

To accommodate texture and noise variability across images, for each image we aggregate the feature sequences of all contours $\bar{k}$, R, N and determine data-driven thresholds via empirical quantiles:

(21) $$T_{k}=Q_{0.75}\left(\bar{k}\right),T_{R}=Q_{0.85}\left(R\right),T_{N}=Q_{0.75}\left(N\right)$$

These thresholds are computed separately for each image from its own contour statistics, and the same quantile settings are used across all scenes and both the public and Xinjiang subsets, so no site-specific manual tuning is required.

After obtaining the three indicators (mean curvature $\bar{k}$, perimeter–area ratio *R*, and noise level *N*), each contour $C_{i}$ is assigned to one of three categories according to its position relative to the thresholds $T_{k}$, $T_{R}$, and $T_{N}$. Contours with low curvature, low perimeter–area ratio, and low noise $\left(\overset{ˉ}{k}\leq T_{k},R\leq T_{R},N\leq T_{N}\right)$ are treated as regular. Contours whose noise remains low $\left(N\leq T_{N}\right)$ but whose curvature or perimeter–area ratio exceeds the threshold $\left(\overset{ˉ}{k}>T_{k}\;\mathrm{or}\;R>T_{R}\right)$ are treated as complex. Contours with high noise $\left(N>T_{N}\right)$, regardless of $\overset{ˉ}{k}$ and R, are treated as noisy. Formally, the classification rule can be written as:

(22) $$C\left(C_{i}\right)=\{\begin{array}{c} Regular\;type,\bar{k}\leq T_{k},R\leq T_{R},N\leq T_{N}\\ Complex\;type,\left(\bar{k}>T_{k}\;\mathrm{or}\;R>T_{R}\right)\;\mathrm{and}\;N\leq T_{N}\\ Noisy\;type,N>T_{N} \end{array}$$

In the second step (multi-strategy adaptive simplification), we adopt tailored strategies for different contour types. DP is suited to regular contours; it recursively removes redundant vertices to achieve efficient compression while preserving overall geometry, yielding $M_{DP}$. Visvalingam–Whyatt (VW) [44] is suited to complex contours; it iteratively deletes points with minimal effective area, preserving local concavities and avoiding oversmoothing, yielding $M_{VM}$. Curvature-based adaptive sampling is suited to noisy contours [2]; it preserves high-curvature regions while smoothing high-frequency noise, yielding $M_{Curv}$.

Since a single algorithm rarely suits all shapes, we design a weighted scoring function:

(23) $$S\left(M^{\prime }\right)=w_{1}f_{k}\left(M^{\prime }\right)+w_{2}f_{R}\left(M^{\prime }\right)+w_{3}f_{N}\left(M^{\prime }\right)$$

where $f_{k}$ measures curvature preservation fitness, that is, the agreement of curvature distributions before and after simplification; $f_{R}$ measures the rationality of the perimeter to area ratio, reflecting the maintenance of overall shape complexity; and $f_{N}$ measures noise suppression, reflecting the removal of spurs and high-frequency perturbations. The weights $\left(w_{1},w_{2},w_{3}\right)$ are set empirically to balance these criteria. The final selection is:

(24) $$\hat{M}=arg\underset{M^{\prime }\in \left\{M_{DP},M_{VM},M_{Curv}\right\}}{\max}S\left(M^{\prime }\right)$$

that is, choose the candidate with the maximum score. This adaptive selection balances regularity, complexity, and noise sensitivity across contour types, improving robustness and generality.

In the third step (multi-directional regularization), even after simplification, edges may be misaligned or corner angles drift. We therefore further regularize by line detection, directional clustering, and edge normalization. For line detection, boundary points $\left(x,y\right)$ are expressed in polar space and the Hough transform is applied:

(25) p=xcosθ+ysinθ

where *p* is the distance to the origin and θ is the line orientation. Directional clustering is then performed by applying *k*-means to the detected θ values to obtain a set of principal directions $\left\{\theta_{m}\right\}$. For edge normalization, each edge orientation

(26) $$\theta_{i}=\tan^{-1}2\left(y_{i+1}-y_{i},x_{i+1}-x_{i}\right)$$

is projected to its nearest principal direction $\theta_{m}$, ensuring that edges are parallel or orthogonal to dominant structural directions.

In the final step (topology checks and optimization), we perform topological validity checks: (1) self-intersection, to ensure that there are no self-intersections or repeated edges; otherwise the polygon is split or adjusted; (2) area check, to avoid zero area or degenerate polygons; and (3) closure, to ensure a unique closure in which the first and last vertices coincide. If any check fails, we roll back and adjust parameters (for example, reduce the simplification thresholds and retain more vertices) until all checks are passed. The resulting building outlines are smooth, compact, and topologically valid, which reduces redundancy while preserving the principal shape and geometry, and provide high-quality vector data for downstream applications.

As a post hoc stage, this pipeline is applied after the network has produced a binary building mask and operates only on contour vertices rather than on the full 500 × 500 raster grid, so its computational cost grows approximately linearly with the total number of vertices and remains modest compared with the CNN–SSM forward pass in our experiments.

## 3. Experiments and Results

### 3.1. Datasets and Experimental Setup

We constructed the experimental dataset from two types of data. The first consists of the Chinese Typical Urban Buildings dataset [45], which covers the central districts of Beijing, Shanghai, Shenzhen, and Wuhan and was acquired from 2017 to 2019 at a spatial resolution of 0.29 m, with tiles of 500 × 500 pixels. The second consists of a self-constructed Xinjiang buildings dataset, annotated using the same protocol as the public dataset to increase regional and scene diversity. Across the 600 tiles, individual images range from a few isolated buildings to dense blocks with many rooftops; most images contain dozens of footprints, providing both sparse and crowded layouts. In practice, the public subset primarily includes dense high-rise urban cores in the four cities, whereas the Xinjiang subset supplements the dataset with lower-density residential and industrial areas at the desert fringe, thereby increasing background diversity (e.g., bright bare soil and sparse vegetation). Combined, the dataset contains 600 images, each with pixel-level building masks.

For the public subset, we directly use the official building masks provided with the dataset; for the Xinjiang subset, building masks are manually annotated from the orthoimages following the same labeling protocol, and all annotations are subsequently reviewed and corrected by a second annotator to ensure consistent labeling quality across both subsets. The data were partitioned into training, validation, and test sets in an 8:1:1 ratio, resulting in 480, 60, and 60 images, respectively. Although this composition increases diversity in building types and backgrounds, all images are acquired over Chinese cities; thus, the reported results should be interpreted primarily as an in-distribution evaluation rather than a cross-region transfer study.

We use a GPU-accelerated environment with an NVIDIA Tesla V100 (NVIDIA Corporation, Santa Clara, CA, USA; 32 GB). The software stack is built via Docker on Ubuntu 22.04.1, and models are implemented with PyTorch (v1.11.0).

We next present the experimental protocol, covering parameter search and loss optimization, ablation configurations, comparisons of post-processing strategies, baseline benchmarking, and the evaluation metrics and complexity indicators used in this study.

(1) Parameter search and loss optimization.

Using HRNet as the base, we jointly grid search learning rate and loss weights to obtain the optimal configuration. Learning rates (1 × 10^−5^, 1 × 10^−4^, 1 × 10^−3^) are tested with Adam, for 100 epochs and batch size = 16. The objective is GridSearchCombinedLoss. Validation is conducted on intersection over union (IoU), with F1-score monitored, to determine the best LR and weight combination.

(2) Ablation on network structure.

To evaluate the contribution of each component, we design four ablations:

Stem: replace the original HRNet stem with the filtering-enhanced module to highlight boundaries and suppress noise;

Stage 1 (Involution): replace standard residual units with Involution residual blocks to enhance local geometric modeling;

Stage 3 (Mamba branch): add a Mamba-SSM global branch whose Selective Scan models cross-scale long-range dependencies with linear complexity, improving global consistency and robustness in complex scenes;

Full model (building model): integrate all three—enhanced stem, Involution, and Mamba—forming the complete HR-Mamba framework.

In all ablation variants, Stage 2 remains identical to the original HRNet design and is not altered, so we do not report a separate Stage 2 modification.

For fair comparison, all ablation variants are trained with the proposed GridSearchCombinedLoss, using the grid-searched optimal weights identified in Section 3.2 under identical training settings and the same 100-epoch schedule without early stopping.

(3) Comparative Experiments on Post-Processing Methods.

To assess the geometric contribution of the post-processing stage, we compare four controls against our method on the same masks: DP simplification only; regularization only (principal direction extraction and edge normalization); non-adaptive simplification followed by regularization; and our adaptive pipeline (thresholds derived from curvature, perimeter to area ratio, and a global noise metric to classify contours into regular, complex, and noisy, followed by DP, VM, or curvature-based sampling, multi-directional regularization, and topological checks).

(4) Model comparison.

To verify effectiveness, we compare against U-Net, HRNet, and Swin Transformer under identical data and training settings. The proposed HR-Mamba with geometric post-processing achieves the best IoU, F1-score, boundary fineness, and small-object retention, demonstrating superiority and robustness in complex urban scenes.

(5) Evaluation metrics.

We adopt four common segmentation metrics, Precision, Recall, F1-score, and IoU:

(27) $$\mathrm{Precision}=\frac{\mathrm{TP}}{\mathrm{TP}+\mathrm{FP}}$$

(28) $$\mathrm{Recall}=\frac{\mathrm{TP}}{\mathrm{TP}+\mathrm{FN}}$$

(29) $$F1\text{-}\mathrm{score}=\frac{2\mathrm{TP}}{2\mathrm{TP}+\mathrm{FN}+\mathrm{FP}}$$

(30) $$\mathrm{IoU}=\frac{\mathrm{TP}}{\mathrm{TP}+\mathrm{FP}+\mathrm{FN}}$$

Here, TP is true positives, FP false positives, FN false negatives. Precision gauges prediction correctness, Recall measures positive coverage, F1-score balances them, and IoU directly reflects region overlap.

In this study, the metrics reported in Table 1, Table 2 and Table 3 are first computed from a single confusion matrix aggregated over all pixels of the 60 test images, corresponding to a pixel-level micro-averaging scheme on the test set.

In addition, to analyze the variability across test images, we also compute the metrics on a per-image basis. Let ${\mathrm{Precision}}_{t}$, ${\mathrm{Recall}}_{t}$, ${F1\text{-}\mathrm{score}}_{t}$, and ${\mathrm{IoU}}_{t}$ denote the metrics computed from the confusion matrix of the t-th test image $\left(t=1,\ldots ,N\right)$. For each metric $m_{t}\in \left\{{\mathrm{Precision}}_{t},{\mathrm{Recall}}_{t},{F1\text{-}\mathrm{score}}_{t},{\mathrm{IoU}}_{t}\right\}$, the mean and standard deviation over the test set are calculated as:

(31) $$\overset{ˉ}{m}=\frac{1}{N}\sum_{t=1}^{N}m_{t}$$

(32) $$s_{m}=\sqrt{\frac{1}{N-1}\sum_{t=1}^{N}{\left(m_{t}-\overset{ˉ}{m}\right)}^{2}}$$

where $\overset{ˉ}{m}$ is the mean and $s_{m}$ is the sample standard deviation. The per-image mean ± standard deviation for all models is summarized in Table 4.

Beyond accuracy, we also assess model complexity and efficiency:

Number of parameters: reflects trainable weights; fewer parameters ease storage/deployment, whereas more parameters may improve expressiveness but can risk overfitting.

FLOPs: floating-point operations per forward pass; a key indicator of computational complexity and inference efficiency. Lower FLOPs suit resource-constrained deployment, while higher FLOPs often indicate stronger capacity at higher cost.

Accordingly, HR-Mamba is evaluated along two axes: segmentation accuracy (Precision, Recall, F1, IoU) and complexity (parameters, FLOPs), ensuring both high extraction accuracy and practical deployability.

### 3.2. Results

(1) Parameter search and loss optimization.

We jointly grid search over learning rates lr ∈ (1 × 10^−5^, 1 × 10^−4^, 1 × 10^−3^) and GridSearchCombinedLoss weights α ∈ (0.4, 0.5, 0.6) (BCE), β ∈ (0.2, 0.3, 0.4) (Dice), γ ∈ (0.0, 0.1, 0.2) (Boundary). This yields 3×3×3×3=81 configurations in total, obtained by taking every combination of one value from each set. All other training settings (optimizer, batch size, number of epochs, and data splits) are kept fixed, so this grid search can be exactly reproduced by enumerating these 81 configurations.

**Figure 6:**
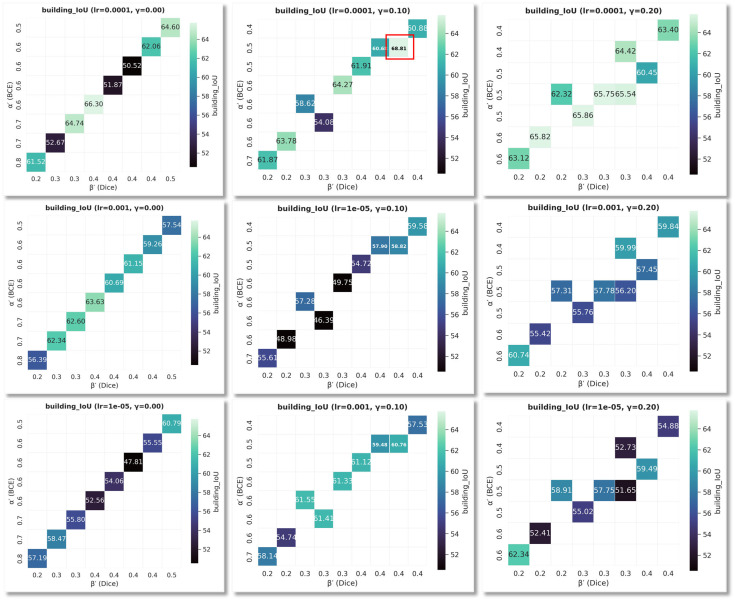
Parameter-search heatmap (Panels are lr and γ; horizontal axis: β; vertical axis: α; color: building_IoU).

The validation set uses IoU as the primary metric (F1 monitored). Results are visualized as a heatmap (Figure 6): rows are learning rates; columns are γ; the horizontal axis is β; the vertical axis is α; color encodes building_IoU.

Learning rate. lr = 1 × 10^−4^ outperforms 1 × 10^−3^ and 1 × 10^−5^ for most weight combinations, indicating better convergence stability and generalization; too large oscillates, too small underconverges.

Boundary weight γ Moderate boundary constraints substantially improve IoU—γ = 0.1 is best; γ = 0 lacks boundary learning; γ = 0.2 shows mild degradation under some LRs, suggesting overly strong boundary terms may compromise regional consistency.

Synergy of α and β. The best/second-best combinations cluster at α∈ [0.5, 0.6], β∈ [0.3, 0.4], indicating pixelwise classification (BCE) and region overlap (Dice) should remain relatively balanced; overly favoring either hinders overall gains.

Optimal configuration and numbers. At lr= 1 × 10^−4^, γ = 0.10, α = 0.50, β = 0.40, we obtain the global optimum, building_IoU = 68.81% (red box in the heatmap). This configuration also shows consistent F1 advantages and is adopted as the default in subsequent ablations and comparisons.

**Figure 7:**
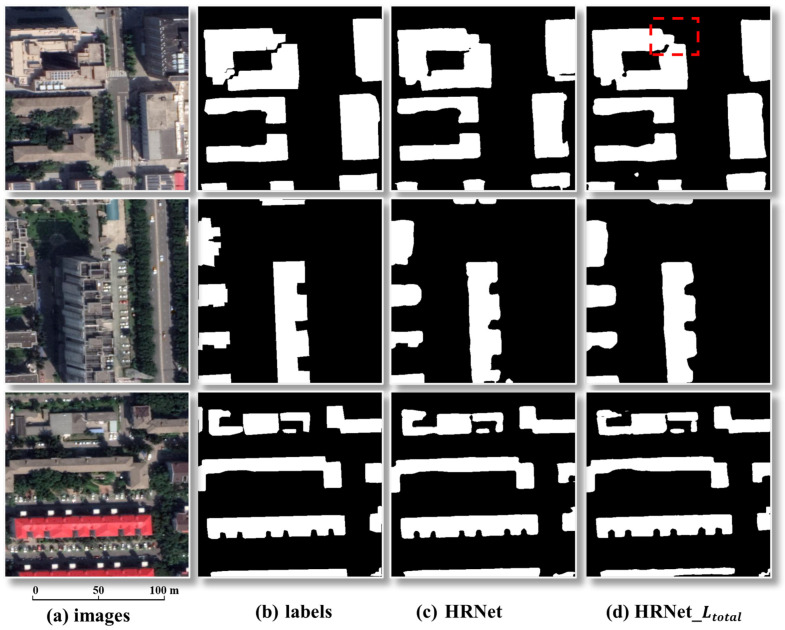
Prediction results for different loss functions. Note: We report results for two loss configurations: HRNet (original loss) and HRNet_$L_{total}$ (GridSearchCombinedLoss). The red dashed box highlights a representative region where HRNet_$L_{total}$ produces a better building extraction than HRNet.

To assess the effectiveness of the loss optimization, Table 1 reports the metrics and Figure 7 shows the visuals. Compared with HRNet (original loss), HRNet_$L_{total}$ (GridSearchCombinedLoss) yields consistent gains on the two key metrics: F1 improves from 80.95% to 81.52%, and IoU increases from 68.05% to 68.81%gei. As highlighted by the red boxes in Figure 6, HRNet_$L_{total}$ (GridSearchCombinedLoss) produces markedly cleaner boundary delineation: façade edges are straighter, corners are sharper and closer to right angles, narrow inter-building gaps are correctly preserved, and thin structures or small attachments are no longer eroded or broken.

(2) Ablation on network structure.

Under a unified training setup with the proposed GridSearchCombinedLoss, we conduct a sequential ablation toward HR-Mamba by starting from the HRNet baseline and progressively adding CM_stem, Involution, and Mamba (Table 2). Relative to the baseline, CM_stem increases F1-score by 0.81% and IoU by 1.15%, with parameters unchanged and FLOPs increasing by 0.01%. Involution increases F1-score by 0.73% and IoU by 1.05%, while parameters decrease by 0.19% and FLOPs decrease by 2.30%. This reduction arises because the Involution block replaces two dense 3 × 3 convolutions in Stage 1 with an input-adaptive kernel that shares weights across channel groups and uses a bottleneck kernel generator, leading to fewer multiply–accumulate operations overall. Mamba delivers larger single-module gains, increasing F1-score by 1.66% and IoU by 2.40%, with parameters increasing by 0.24% and FLOPs increasing by 0.28%. Combining all three, HR-Mamba achieves the best results, increasing F1-score by 2.05% and IoU by 2.97%, with parameters increasing by only 0.04% and FLOPs decreasing by 2.00%. This sequential ablation demonstrates complementary contributions: F1-score and IoU improve markedly while model size remains virtually unchanged and computational cost is balanced or reduced.

**Figure 8:**
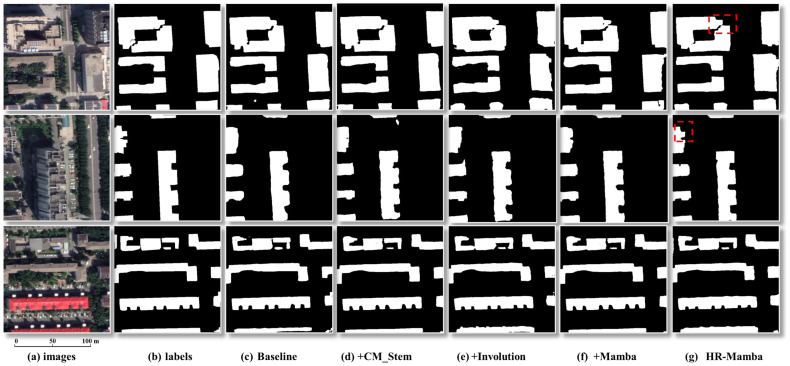
Qualitative ablation results toward HR-Mamba. Note: From left to right: Images, Labels, Baseline, +CM_stem, +Involution, +Mamba, HR-Mamba. The red boxes highlight regions for boundary/detail comparison; only HR-Mamba recovers the correct shape and boundary, whereas other ablated variants fail to do so.

As shown in Figure 8, adding CM_stem, Involution, and Mamba progressively improves overall segmentation quality; however, in the red-boxed regions, all ablated variants except HR-Mamba exhibit adhesion, gaps, or contour drift, failing to recover both shape integrity and boundary regularity simultaneously. In contrast, HR-Mamba consistently breaks false bridges, preserves narrow gaps and thin structures, and regularizes corners, yielding contours that align more faithfully with the ground truth and evidencing the synergy between local geometric modeling and global consistency in our design.

(3) Comparative Results of Post-processing Methods.

**Figure 9:**
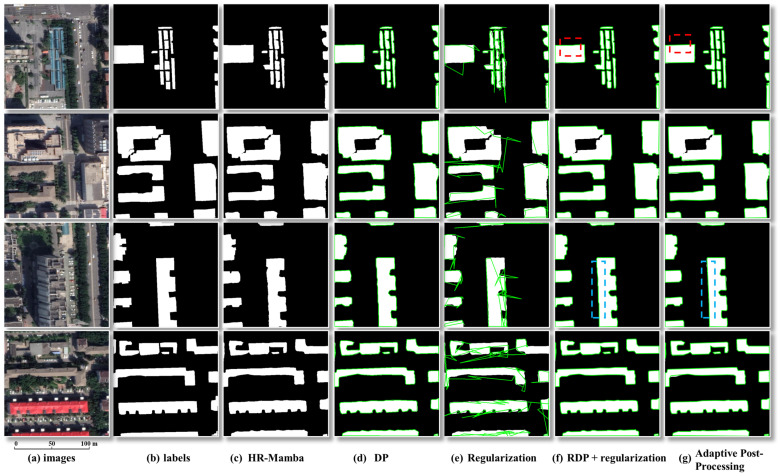
Post-processing ablation: effects of different contour regularization schemes. Note: Green polylines denote the vectorized building boundaries. Columns (left to right): images, labels, HR-Mamba, DP only, regularization only, DP followed by regularization (non-adaptive), and the adaptive method (ours).

As shown in Figure 9, simplification alone yields limited improvement because the green vectors still track jagged raster edges. Regularization alone, without prior noise suppression, amplifies local errors into sharp corners and chaotic zigzags, producing geometric distortion. The non-adaptive pipeline that simplifies first and then regularizes offers partial relief but lacks strategies tailored to regular and complex contours, which leads to underregularization or overregularization. In the regions marked by red boxes, all three baselines fail to recover the correct shape and boundary. At the corresponding locations highlighted by the blue dashed boxes, the adaptive method produces straight façades, near right angle corners, preserved narrow gaps, and no self-intersections or degenerate edges. This outcome accords with the design. Contours are first classified using mean curvature, the perimeter to area ratio, and a global noise index. The pipeline then applies DP, VW, and curvature-based adaptive sampling to regular, complex, and noisy shapes, respectively, followed by principal direction clustering, multi-directional regularization, and topological consistency checks, yielding cleaner and more reliable building boundaries across diverse scenes. For completeness, the measured runtime of the adaptive post-processing on the test set is 3.451 s for 60 tiles (average 0.058 s per image).

(4) Comparative Analysis of Different Models.

Under the standardized evaluation in Table 3, HR-Mamba_adaptive post-processing (APp) attains the best performance across all models, with Recall 0.7949, Precision 0.8875, F1-score 0.8393, and IoU 0.7197. The competing methods include widely used CNN- and Transformer-based segmentation networks, such as U-Net, DeepLabv3+, DANet, SegFormer, HRNet, and Swin Transformer, which form a representative set of baselines for building extraction pipelines. Recently proposed Mamba-based networks for remote sensing semantic segmentation, such as the Vision-Mamba-based CVMH-UNet and the multi-modal MFMamba [31,32], are discussed in the Introduction but are not included as separate baselines here, because this work focuses on integrating a Mamba-SSM branch into an HRNet-style high-resolution backbone and coupling it with geometry-driven vector post-processing for building footprints.

Relative to the HRNet baseline, it increases Recall by 2.19%, Precision by 3.88%, F1-score by 2.98%, and IoU by 3.92%, while being more compute-efficient: FLOPs 89.81 G (2.00% lower than HRNet) and parameters 66.40 M (increase of 0.04%). Compared with Swin Transformer, HR-Mamba_APp delivers higher F1-score by 2.39% and IoU by 3.01% with approximately 57% fewer FLOPs. Compared with SegFormer, it uses approximately 22% more FLOPs but achieves substantially higher F1-score by 6.59% and IoU by 9.15%. Overall, HR-Mamba_APp provides a superior balance between accuracy and efficiency, yielding markedly better boundary fidelity and region-overlap quality with a model size close to HRNet and substantially lower computation than heavy self-attention models.

**Figure 10:**
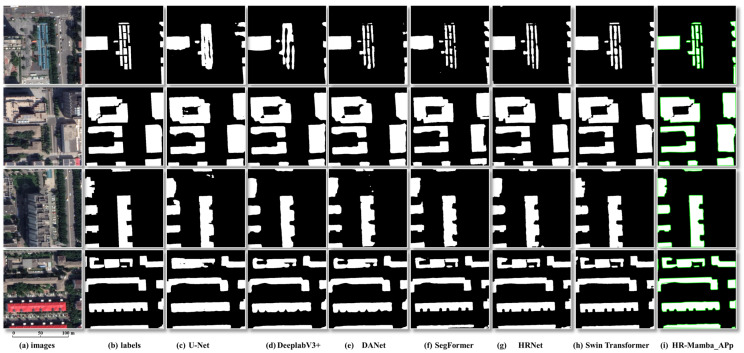
Visual comparison of different models for building extraction (with adaptive post-processing). Note: Columns from left to right: images, labels, U-Net, DeepLabv3+, DANet, SegFormer, HRNet, Swin Transformer, HR-Mamba_APp. The green contours highlight the building outlines obtained by HR-Mamba with adaptive post-processing (HR-Mamba_APp).

As shown in Figure 10, U-Net and DeepLabv3+ tend to produce false bridges, holes, and noisy contours, leading to weak shape recovery. DANet moderately improves global consistency but still exhibits adhesion and corner drift between adjacent buildings. SegFormer and HRNet cover the main regions more completely, yet narrow gaps and small components are often smoothed out and the boundaries remain irregular. Swin Transformer is stable on large structures, but boundary jitter and rounded right angles persist under shadows and complex textures. In contrast, HR-Mamba_APp simultaneously enhances regional consistency and geometric regularity: narrow gaps are preserved, adjacent roofs are separated, façades are straighter, corners are closer to right angles, and the green vector boundaries remain spur-free and topologically valid. These qualitative findings are consistent with Table 3 and further underline the superiority of HR-Mamba_APp over Swin Transformer in boundary stability and shape fidelity.

## 4. Discussion

The combination of learning-based segmentation and rule-based geometric regularization is a central strength of HR-Mamba. By letting the CNN with SSM fusion perform the heavy semantic parsing, the model attains high recall and strong overall accuracy. The subsequent geometry-driven post-processing enforces shape constraints that reflect building orthogonality, parallel edges, and alignment, producing polygons that are directly usable for mapping. Quantitatively, comparing HR-Mamba before and after adaptive post-processing (HR-Mamba_APp) isolates the effect of this stage: F1-score increases from 83.57% to 83.93% and IoU from 71.78% to 71.97% (Table 2), indicating that, overall, the geometry-driven refinement corrects more segmentation errors than it introduces while only slightly perturbing the underlying masks. This explicitly distinguishes HR-Mamba from existing CNN- and Transformer-based building extraction networks, which typically focus on raster masks and do not explicitly couple high-resolution global modeling with a geometry-driven vector regularization pipeline for building footprints. This hybrid design addresses a common critique of pure deep learning in remote sensing, namely that pixelwise-accurate masks can still be impractical because of irregular outlines or minor violations of cartographic rules.

**Figure 11:**
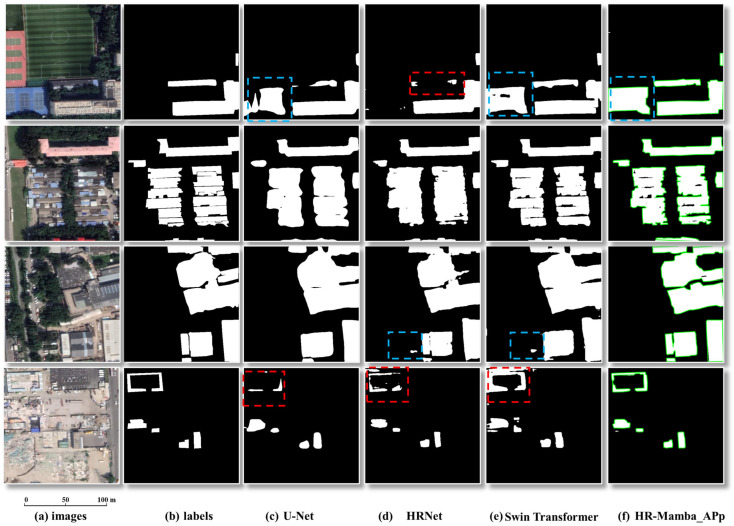
Typical failure modes of baseline models and HR-Mamba_APp in dense urban scenes. From left to right: images, labels, U-Net, HRNet, Swin Transformer, and HR-Mamba_APp. The green contours highlight the building outlines obtained by HR-Mamba with adaptive post-processing (HR-Mamba_APp).

To make the remaining limitations more explicit, Figure 11 visualizes typical failure modes for U-Net, HRNet, Swin Transformer, and HR-Mamba_APp on four dense urban patches. Red dashed boxes highlight small buildings or building parts that are missed by the networks, whereas blue dashed boxes mark false positives where non-building linear structures are classified as buildings. In the first-row example, the blue-boxed elongated strip is visually similar to a rooftop from a nadir view, so even human observers may reasonably mistake it for a building. Compared with the baselines, HR-Mamba_APp largely eliminates adhesion and jagged edges and recovers more small targets, but very low-contrast buildings and ambiguous background objects can still lead to omissions or occasional oversegmentation, illustrating that some errors remain intrinsically difficult at the image level.

A practical consideration is the scope of the regularization prior. Our procedure assumes predominantly straight edges, which holds for most urban and residential buildings but can be suboptimal for circular or organic structures. Such cases were rare in our test sets. The pipeline can be made adaptive by detecting roundness or low directional consensus and skipping or relaxing regularization for those instances. A further extension is to couple geometry regularization with training through a differentiable proxy loss or learned polygonization, which can improve end-to-end consistency at the cost of a more complex optimization.

From a computational perspective at 500 × 500 resolution, the Mamba-SSM fusion remains efficient in terms of model size and operations. Relative to HRNet, HR-Mamba increases the parameter count by 27,960, which represents an increase of 0.0421% over the baseline, and reduces FLOPs by 1.83 G, which represents a reduction of 1.9969% relative to the baseline (see Table 2 and Table 3). These results show that global-dependency modeling can be introduced with negligible growth in model size and lower computational cost. In addition, the geometry-driven post-processing is implemented as a lightweight routine on the extracted polygons and, under the same 500 × 500 tile setting, does not dominate the end-to-end runtime. Consistently, the measured average post-processing time is approximately 0.058 s per 500 × 500 tile, corresponding to a total of 3.451 s over 60 tiles.

Beyond the micro-averaged metrics obtained from the aggregated confusion matrix on the test set, we further assess the variability of the results across individual images. Table 4 reports the per-image mean ± standard deviation of Precision, Recall, F1-score, and IoU for all models on the 60 test images. As shown in Table 4, the relative ranking of the models remains unchanged when per-image statistics are considered, and HR-Mamba_APp still achieves the highest mean F1-score and IoU. The improvements of HR-Mamba_APp over HRNet and Swin Transformer are comparable to or larger than the corresponding per-image standard deviations, indicating that the observed 2–3% gains are consistently reflected across the test images rather than being driven by a few isolated samples.

A limitation of the present study is that our experimental benchmark does not include head-to-head comparisons against other Mamba-based architectures. While several Vision-Mamba-based and Mamba-based networks have recently been proposed for remote sensing semantic segmentation [31,32], training and tuning all these models on our combined building dataset lies beyond the scope of this work. Extending the evaluation to a broader family of Mamba-based backbones on unified building extraction benchmarks is an important direction for future research and will help further contextualize the performance of HR-Mamba.

Typical residual errors fall into two categories. First, extremely small buildings, such as sheds below 10 m^2^, can be missed when their appearance is weak. Second, very complex facilities with narrow connectors may be partially split, sometimes reinforced by regularization that prefers clean separations at thin links. Many mapping guidelines accept separate footprints unless buildings are truly attached, but this behavior highlights a limitation: attachments and shared walls are not modeled explicitly. Future work can incorporate instance-merging logic, structured reasoning about adjacency, or a panoptic formulation to handle attachments more reliably.

Beyond model design, there are broader avenues for improving generalization and transferability. The current training emphasizes dense high-rise urban scenes; adding rural and suburban samples as well as cross-region imagery from other countries should improve robustness to greater appearance diversity. Rural-specific cues, such as road-network priors or targeted attention mechanisms for contiguous single-story houses and farm structures, may further raise accuracy. Finally, fusing additional modalities, including SAR, LiDAR point clouds, and GIS vectors, is a promising direction to improve both accuracy and robustness. We also see value in combining HR-Mamba with domain-adaptation or domain-generalization techniques to better handle changes in acquisition conditions and building styles. We will pursue these directions while maintaining engineering-ready outputs, namely regular and topologically valid building polygons.

## 5. Conclusions

Conventional building segmentation methods and plain convolutional neural networks have limited ability to capture fine-grained geometry and boundary consistency in complex urban scenes, often leading to missed or false detections of small buildings in dense areas and to boundary adhesion between adjacent buildings, which hinders precise segmentation. Building on an improved HRNet, we develop HR-Mamba by adding several complementary components. In this work, we build on a high-resolution HRNet backbone and develop HR-Mamba, which combines an edge-aware Canny–median stem, Involution-based residual blocks for spatially adaptive local feature extraction, a Mamba-SSM global branch for linear-time long-range dependency modeling, a grid-searched composite loss (BCE, Dice, Boundary), and a geometry-driven adaptive post-processing framework that produces and topologically valid building polygons.

On dense urban imagery, HR-Mamba_APp attains an F1-score of 83.93% and an IoU of 71.97% relative to HRNet’s 80.95% and 68.05%; while keeping the parameter count nearly unchanged and slightly reducing FLOPs. Under the same training and evaluation protocol, HR-Mamba_APp also surpasses representative CNN and Transformer baselines in both overlap-based and boundary-sensitive metrics, and qualitative results show cleaner building contours, better separation of adjacent buildings, and fewer artifacts in narrow gaps and corners.

Overall, HR-Mamba demonstrates that low-level edge stabilization, position-adaptive local operators, linear-time global reasoning, and lightweight geometry regularization within a high-resolution backbone can deliver GIS-ready building polygons at practical computational cost. Although most individual components are adapted from existing ideas, their integration into a unified high-resolution pipeline tailored for building footprint extraction yields a robust, engineering-oriented solution for complex urban scenes and suggests a general template for coupling state space models with geometry-aware post-processing in remote sensing segmentation.

## Figures and Tables

**Table 1:** Evaluation metrics for different loss functions.

Model	Recall	Precision	F1-Score	IoU
HRNet	0.7730	0.8487	0.8095	0.6805
$\mathrm{HRNet}\text{\_}L_{total}$	0.7745	0.8601	0.8152	0.6881

**Table 2:** Evaluation metrics for different ablations.

Model	Recall	Precision	F1-Score	IoU	Params	Flops
Baseline	0.7745	0.8601	0.8152	0.6881	66,367,684	91.64 G
+CM_stem	0.7852	0.8652	0.8233	0.6996	66,367,684	91.65 G
+Involution	0.7863	0.8623	0.8225	0.6986	66,238,732	89.53 G
+Mamba	0.7922	0.8756	0.8318	0.7121	66,524,596	91.90 G
HR-Mamba	0.7953	0.8804	0.8357	0.7178	66,395,644	89.81 G

**Table 3:** Evaluation metrics for different models.

Model	Recall	Precision	F1-Score	IoU	Params	FLOPs
U-Net	0.6521	0.6431	0.6475	0.4782	29,060,676	58.12 G
DeepLabv3+	0.6674	0.6522	0.6597	0.4914	41,237,482	119.7 G
DANet	0.7395	0.7764	0.7575	0.6106	29,060,676	281.10 G
SegFormer	0.7532	0.7948	0.7734	0.6282	81,970,370	73.54 G
HRNet	0.7730	0.8487	0.8095	0.6805	66,367,684	91.64 G
Swin Transformer	0.7810	0.8524	0.8154	0.6896	59,840,992	210.78 G
HR-Mamba_APp	0.7949	0.8875	0.8393	0.7197	66,395,644	89.81 G

**Table 4:** Per-image mean ± standard deviation of evaluation metrics on the test set (60 images).

Model	$\mathrm{Recall}\;(\pm s_{m}$)	$\mathrm{Precision}\;(\pm s_{m}$)	$F1\text{-}\mathrm{Score}\;(\pm s_{m}$)	$\mathrm{IoU}\;(\pm s_{m}$)
U-Net	0.6521 ± 0.1325	0.6431 ± 0.1402	0.6475 ± 0.1360	0.4782 ± 0.1503
DeepLabv3+	0.6674 ± 0.1289	0.6522 ± 0.1367	0.6597 ± 0.1321	0.4914 ± 0.1478
DANet	0.7395 ± 0.1103	0.7764 ± 0.1055	0.7575 ± 0.1072	0.6106 ± 0.1210
SegFormer	0.7532 ± 0.1027	0.7948 ± 0.0978	0.7734 ± 0.0995	0.6282 ± 0.1159
HRNet	0.7730 ± 0.0951	0.8487 ± 0.0897	0.8095 ± 0.0910	0.6805 ± 0.1045
Swin Transformer	0.7810 ± 0.0938	0.8524 ± 0.0885	0.8154 ± 0.0891	0.6896 ± 0.1012
HR-Mamba_APp	0.7949 ± 0.0892	0.8875 ± 0.0830	0.8393 ± 0.0845	0.7197 ± 0.0958

## Data Availability

The public dataset used in this study is available at the following DOI: https://doi.org/10.11922/sciencedb.00620. The Xinjiang regional subset is an author-curated extension subject to data-use restrictions, so the full raw dataset cannot currently be released in a public repository. However, the tiles used in our experiments (image patches, pixel-level building masks, and the indices defining the training, validation, and test splits) are available from the corresponding author for non-commercial research upon reasonable request. Interested researchers may contact the corresponding author with a brief description of the intended research purpose.
